# Functional Androdioecy in Critically Endangered *Gymnocladus assamicus* (Leguminosae) in the Eastern Himalayan Region of Northeast India

**DOI:** 10.1371/journal.pone.0087287

**Published:** 2014-02-28

**Authors:** Baharul Islam Choudhury, Mohammed Latif Khan, Selvadurai Dayanandan

**Affiliations:** 1 Forest and Evolutionary Genomics Laboratory, and Centre for Structural and Functional Genomics, Biology Department, Concordia University, Quebec, Canada; 2 Québec Centre for Biodiversity Sciences, Montréal, Quebec, Canada; 3 Department of Forestry, North Eastern Regional Institute of Science & Technology, Nirjuli, Arunachal Pradesh, India; University of Massachusetts, United States of America

## Abstract

*Gymnocladus assamicus* is a critically endangered tree species endemic to Northeast India, and shows sexual dimorphism with male and hermaphrodite flowers on separate trees. We studied phenology, reproductive biology and mating system of the species. The flowers are small, tubular, odorless and last for about 96 hours. Pollen grains in both morphs were viable and capable of fertilization leading to fruit and seed set. Scanning electron micrographs revealed morphologically similar pollen in both male and hermaphrodite flowers. The fruit set in open pollinated flowers was 43.61 percent, while controlled autogamous and geitonogamous pollinations yielded 76.81 and 65.58 percent fruit set respectively. Xenogamous pollinations between male and hermaphrodite flowers resulted in 56.85 percent fruit set and pollinations between hermaphrodite flowers yielded 67.90 percent fruit set. This indicates a functionally androdioecious mating system and pollination limited fruit set in *G. assamicus*. Phylogenetic analyses of *Gymnocladus* and the sister genus *Gleditsia* are needed to assess if the androdioecious mating system in *G. assamicus* evolved from dioecy as a result of selection for hermaphrodites for reproductive assurance during colonization of pollination limited high altitude ecosystems.

## Introduction

The tree genus *Gymnocladus* (Leguminosae) comprises five species [1] distributed in the Eastern North America and Eastern Asia [2], and considered to have originated in the Eastern Asia during the Eocene and migrated across the Bering land bridge to North America [4]. *G. diocus* (L.) K. Koch is restricted to North America, while *G. angustifolius* (Gagnep.) J.E. Vidal is confined to Vietnam. The remaining three species, *G. chinensis* Baill., *G. assamicus* Kanjilal ex P.C. Kanjilal and *G. burmanicus* Parkinson are distributed in the region bordering India, China and Myanmar (Burma). The geographical ranges of *G. assamicus* and *G. chinensis* within India are restricted to the Northeastern states [3]. *G. assamicus* is a critically endangered tree species with declining populations [5]. Extensive field surveys and environmental niche modelling (ENM) studies revealed existence of only a few remnant populations of the species confined to moist areas on hilly slopes and stream banks in the West Kameng and Tawang districts of Arunachal Pradesh [6].

The mating system of the genus *Gymnocladus* has been broadly described as polygamous, unisexual, bisexual or dioecious [7]–[9]. Although *G. chinensis* is considered as a polygamous species [7], no detailed studies on floral biology or the breeding system of *Gymnocladus* species exist. We studied floral biology of *G. assamicus* and discovered an androdioecious mating system where male and hermaphrodite flowers are produced on separate trees. Androdioecy is a rare mating system [10]–[12], and since Darwin's [14] original report on the androdioecy, no reliable evidence for the occurrence of androdioecy was reported until 1922 [13]. To date, about 50 plants and 36 animal species have been described as androdioecious [15]. In contrast, dioecy (occurrence of male and female plants) and gynodioecy (occurrence of female and hermaphrodite plants) are known to occur in approximately 6% [16] and 10% [17] of angiosperms respectively. Since the first confirmed report of androdioecy in *Datisca glomerata* (Datiscaceae) [18], several reports of androdioecy have been published [12], [19]–[22]. During the last two decades, several plant species have been described as androdioecious on the basis of morphology. However, detailed studies have revealed that many such species are functionally dioecious or cryptic dioecy with sterile pollen in morphologically hermaphroditic flowers [23]. Only few plant species including *Datisca glomerata* [18], *Mercurialis annua* [24], *Schizopepon bryoniaefolius* [25] and *Morinda umbellata* subsp. *boninensis* [26] have been confirmed to be functionally androdioecious, where both male and hermaphrodite flowers produce fertile pollen.

In the present study, we demonstrate the functional androdioecy in *Gymnocladus assamicus*, a less known and endangered tree species in the Eastern Himalayan region of Northeast India. We studied the reproductive biology of two plant morphs of *G. assamicus* through (1) quantifying the proportions of male and hermaphrodite plants in populations, (2) analysing their reproductive and vegetative phenology, (3) examining floral features, (4) assessing male and hermaphrodite functions of each sexual phenotype, and (5) determining the breeding system through controlled pollination experiments.

## Materials and Methods

We declare that no specific permissions were required for field studies as study locations were not privately owned or not in protected areas. This study was sponsored by Department of Science and Technology, Government of India (Sanction no. SR/SO/PS-16/2002 to MLK) and no further permission was required.

### Study species

**Figure 1:**
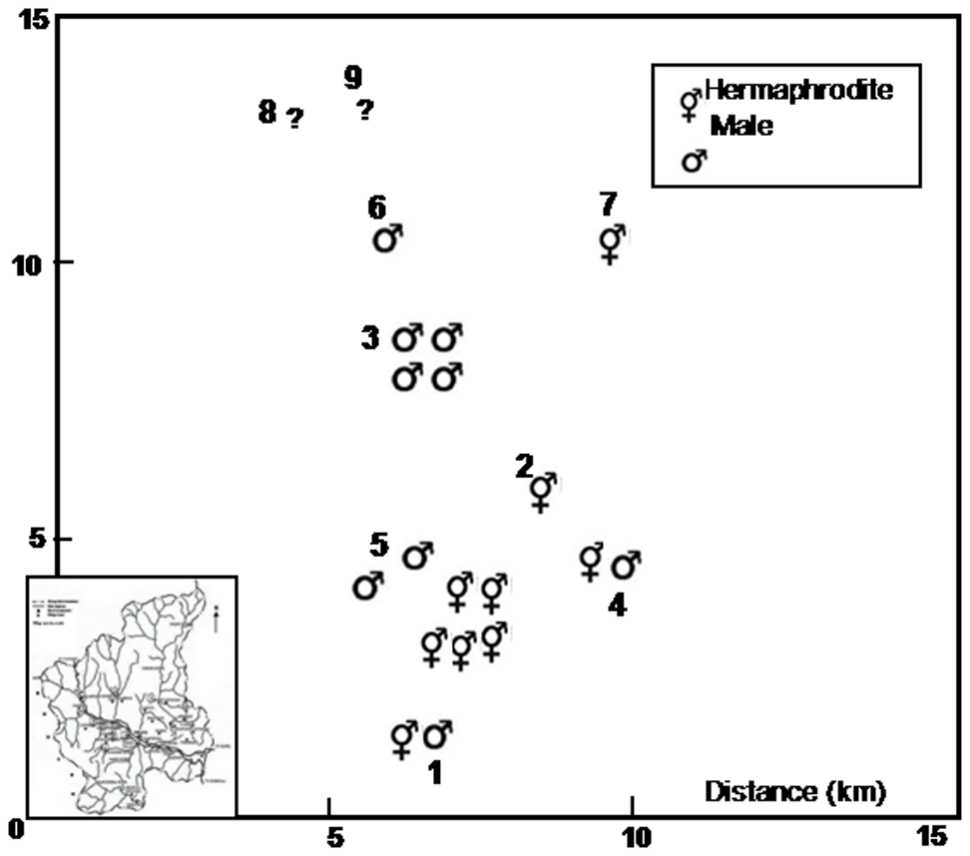
Spatial distribution of male and hermaphrodite trees. **1**- Moishing; **2**- Dirang Village; **3**- Changfu Moon; **4**- Yewang Village; **5**- Dambla Village; **6**- Jyotinagar; **7**- Runkung Village; **8**- Lishpa Village I; **9**- Lishpa Village II.

Since *G. assamicus* is a critically endangered tree species with a declining population size, only a limited number of flowering individuals were available for the present study. The individuals studied were located in high altitudinal (1500–2200 m asl) forests in the village of Dirang (27°15′ to 27°10′N; 92°12′ to 19°14′E) in the West Kameng district of Arunachal Pradesh, India. The climate of the area is subtropical to wet temperate with an annual rainfall of 1752 mm of which 75 percent is normally received during the rainy season between June and October. Average monthly temperature ranges from 0°C during the winter (November–February) to 34°C during the summer (May–June). Out of the total of 28 mature trees included in the present study (Table 1), nine trees were recorded to bear hermaphrodite flowers and produced fruits regularly. The trees were either solitary or occurred as small groups within fragmented forests in an area of about 10 km^2^ (Figure 1). The number of trees in each site ranged from one to six and majority of sites had no seedlings or saplings suggesting a poor regeneration potential of the species.

**Table 1:** Location of *G. assamicus* in and around Dirang.

Site	Locality	No. of individuals		Male/Female ratio
		Male	Bisexual	
MS	Moishing	1	1	1∶1
DB	Dirang Basti	-	1	0∶1
CM	Changfu Moon	4	-	4∶0
YV	Yewang Village	1	1	1∶1
DM	Dambla Basti	2	5	2∶5
JN	Jyotinagar	1	-	1∶0
RV	Runkung Village	-	1	0∶1
LV-I	Lishpa Village I (Rama Camp)	5?		–
LV-II	Lishpa Village II (Rama Camp)	5?		–

(? = unknown).

### Phenology and floral biology

Eighteen trees were regularly monitored for their phenology during the period between 2004–2007 and included in the detailed study of floral biology, while nine individuals from two sites namely Moishing (MS) and Dambla Basti (DM) were used for mating system studies through controlled pollination experiments. Ten individuals in Lishpa Village (LV) I and II did not flower during the study period. Phenological events including bud break, leaf flushing, leaf shedding, flowering, fruiting and fruit set were recorded during the study period. Daily observations were made during the peak flowering period to record various stages of floral development. Subsequent events such as fruit set and fruit maturation were recorded on weekly basis. The number of flowers per inflorescence was counted from 20 randomly selected inflorescences per tree and the morphological details of floral parts were observed using a hand lens. Flower longevity was determined by observing 20 marked flowers per tree from the time of flower opening until wilting. Growth and development of various floral parts were recorded twice daily, at around 0600 hrs and 1800 hrs. The volume of nectar produced (µl) in both floral morphs at various developmental stages was measured using graduated micropipettes. The nectar secretion patterns were studied in 20 randomly selected flowers from 20 bagged inflorescences of each floral morph. Measurements were carried out at 0600 hrs and 1800 hrs daily during the entire lifespan of the flower. The floral morphometric measurements were made using a digital calliper (Mitutoyo Japan).

In the present study, the floral ontogeny was divided into six stages as given in Table 2. The pre-anthesis bud stage with purple perianth and closed tepals ready to open was designated as stage I (time: 0 h). The anthesis initiation and opening of tepals (time: 24 h) was considered as stage 2, and almost open flowers with ca. 15–16 mm wide opening of tepals (time: 48 h) were considered as stage 3. At the stage 4, flowers were fully open, and emerged anther lobes were at the level of the stigma. At this stage pollen grains were highly fertile (58–60 percent), and in hermaphrodite flowers, the stigma was highly receptive (time: 72 h). The stage at which flowers begin to wilt, tepals start to curl and reproductive parts enter the drying phase (time: 96 h) was considered as stage 5. The onset of floral senescence, when pollen fertility ceases and the perianth tubes falls was considered as stage 6.

**Table 2:** Stages of floral development of *G. assamicus.*

Stages	Dimension of flower		Stigma receptivity	Pollen fertility (%)		Nectar volume (µl) (M)	
	Length(mm)	Width(mm)		Male	Bisexual	Male	Bisexual
S1	8.15	2.45	X	X	X	X	X
S2	9.44	2.74	X	X	X	X	X
S3	15.75	3.12	Modest	8–10	10–12	2.5–3	2–2.5
S4	16.52	3.74	Maximum	58–60	58–62	16–18	14–15
S5	16.08	3.86	Weak	6–8	8–10	10–12	9–10
S6	15.98	3.33	X	X	X	X	X

### Flower visitors

The visitors to flowers of trees at MS and DM sites were recorded throughout the day during the peak flowering period. The insect visitors were trapped with sweep nets and classified into general groups such as bees, beetles, moths, and butterflies and further identified using reference manuals and assistance from specialists at the Zoological Survey of India (ZSI) in Itanagar, Arunachal Pradesh. Honeybees were identified to the level of species using taxonomic keys, and bird species were identified using photographs, field observations and consulting ornithological experts of the region (Dr. Anwaruddin Choudhury, Rhino Foundation, Guwahati, India).

### Pollen viability and stigma receptivity test

The pollen viability was assessed as a percentage of pollen grains germinated using the sitting drop culture method [27]. Pollen grains were collected from 10 randomly harvested flowers from different individuals of both morphs at different developmental stages, germinated in Brewbaker and Kwack's medium [28] and counted under a compound microscope. Five replicates each from hermaphrodite and male trees were studied. Pollen was considered viable when clear pollen tube growth was visible under the microscope. Stigma receptivity was tested using H_2_O_2_ following the method of Dafni [29]. Emission of bubbles from the stigma surface at high rate was considered strong receptivity, while slow emission of bubbles was considered as weak stigma receptivity.

### Pollen morphology

The pollen morphology was studied using a scanning electron microscope (SEM). Dried pollen samples were mounted on metal stubs, gold coated and observed under a scanning electron microscope (LEO 1430VP, Karlzeiz, Germany).

### Mating system analyses

Mating system experiments were conducted on nine trees located at MS and DM sites. The following controlled pollination experiments were carried out and percent fruit set was recorded.

- Open pollination – fruit set under natural condition in 302 non-manipulated flowers.
- Spontaneous self pollination – fruit set in 396 unopened flowers bagged for excluding flower visitors.
- Controlled pollination: freshly opened flowers were emasculated using fine forceps and bagged for artificial pollination. The flowers at other developmental stages in each chosen inflorescence were removed before bagging. The controlled pollinations were carried out *in situ* during the stigma receptivity period (Stage 4; Table 2). To determine the autogamous (pollen from the same flower) fruit set, hand pollination was carried out in 143 flowers while that of geitonogamous pollination (pollen from another flower of the same tree) was carried out in 160 flowers. The xenogamous pollination (pollen from a different tree) was carried out between male versus hermaphrodites in 134 flowers and hermaphrodites versus hermaphrodites in 147 flowers.
- Agamospermic and parthenocarpic fruit set was determined using 93 bagged and emasculated hermaphrodite flowers without pollination.

### Equilibrium male frequency in the population

In order to assess the congruence between observed frequency of males and the theoretically expected frequency of males in a population under various levels of selfing (s = 0.1, 0.2, and 0.5) and inbreeding depression [30], we plotted the range of predicted male frequency values against inbreeding depression. We used K values slightly lower (K = 2.5) and higher (K = 3.5) than the observed K value (K = 2.9) based on the overall differences in flower production between male and hermaphrodite trees.

## Results

### Phenology

**Figure 2:**
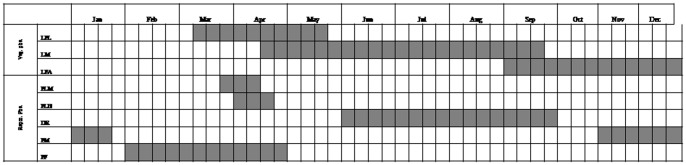
Vegetative and reproductive phenology of male and hermaphrodite *G. assamicus* trees. LFL = Leaf flushing, LM = Leaf maturation, LFA = Leaf fall, FLM = Flowering (Male), FLH = Flowering (Hermaphrodite), DR = Druping, FM = Fruit maturation, FF = Fruit fall.

Individual *G. assamicus* trees remained leafless for over two months during the winter (January–February). The mature pods persisted on trees until the next flowering season. New leaves appeared in early March followed by flowering in April, which lasted for 15–20 days. Phenological patterns of vegetative phases were similar in both plant morphs. Flowers of male trees bloomed between the last week of March and second week of April, whereas blooming of trees with hermaphrodite flowers peaked in April (Figure 2).

### Floral morphology

**Figure 3:**
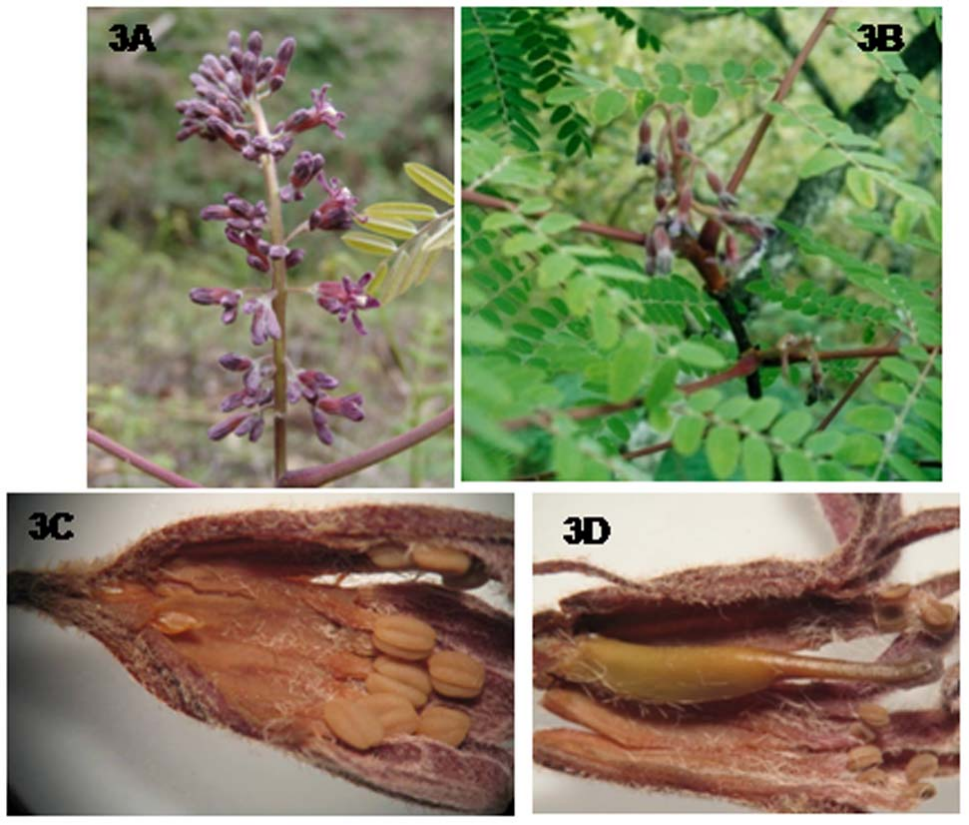
Inflorescence and flower morphology of *G. assamicus*. **A,** Male inflorescence; **B,** hermaphrodite inflorescence; **C,** a male flower showing rudimentary carpel; **D,** a hermaphrodite flower.

Both male and hermaphrodite flowers were borne on terminal racemose inflorescences with fine pubescence, and flowers were tubular in shape, purple in color, odorless and lasted for about 96 hours. Male inflorescences were 13–16 cm long and 5–6 cm wide with nearly whorled lateral branches, which emerged from about 15–20 nodes on the inflorescence axis (Figure 3A; Table 3). Hermaphrodite inflorescences were shorter and ranged from 4–6 cm in length with fewer nodes, and fewer numbers of flowers than male inflorescences (Figure 3B; Table 3). The developmental stages of both male and hermaphrodite flowers were similar and produced similar amounts of nectar (Table 2). The pollen grains of both morphs were viable and fertile.

**Table 3:** Floral characteristics of *G. assamicus.*

Features	Inflorescence Length (cm)	Inflorescence Breadth (cm)	No. of flower per inflorescence	Average flower production	Biomass at fully opened stage (gm)	Length of corolla tube (mm)	Width of corolla tube (mm)	Length of pistil (mm)	Length of anther lobe (mm)	
									Longer	Shorter
Male	12 (±0.81)	2.92 (±0.14)	72.50 (±4.01)	238227.92	0.049 (±0.002)	5.50 (±0.027)	3.43 (±0.038)	--------	8.99 (±0.065)	8.80 (±0.015)
Hermaphrodite	3.38 (±0.11)	2.26 (±0.06)	23.40 (±1.41)	80521.98	0.088 (±0.004)	9.20 (±0.017)	3.74 (±0.014)	10.84 (±0.054)	9.67 (±0.050)	9.07 (±0.025)

±S.E. n = 20.

### Male flowers

Among 28 mature *G. assamicus* trees included in the present study (Table 1), the overall percentage of male trees was 32.14. The male flowers were pedicellate and cylindrical, and male individuals produced as much as 80500 flowers per tree, almost three times than hermaphrodite trees (24000 flowers per hermaphrodite tree; Table 3). The perianth tube of five united tepals was narrow at the base and gradually widened at the tip. Each flower had ten stamens with five longer and five shorter alternatively arranged filaments. Vestigial carpels are visible at the base of the perianth tube (Figure 3C). Male flowers open between 0900–1100 hrs and anthers dehisce after 2000–2400 hrs. Pollen grains collected from freshly opened flowers showed 8–10 percent germination and reached to 58–60 percent germination at the stage 4, and then decreased with the age of the flower reaching to no viability at stage 6 (Table 2).

### Hermaphrodite flowers

**Figure 4:**
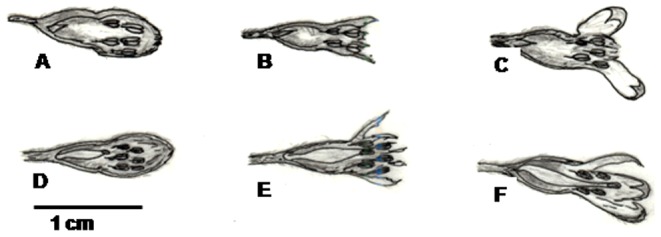
Three developmental stages of male (A, B, C) and hermaphrodite (D, E, F) flowers.

**Figure 5:**
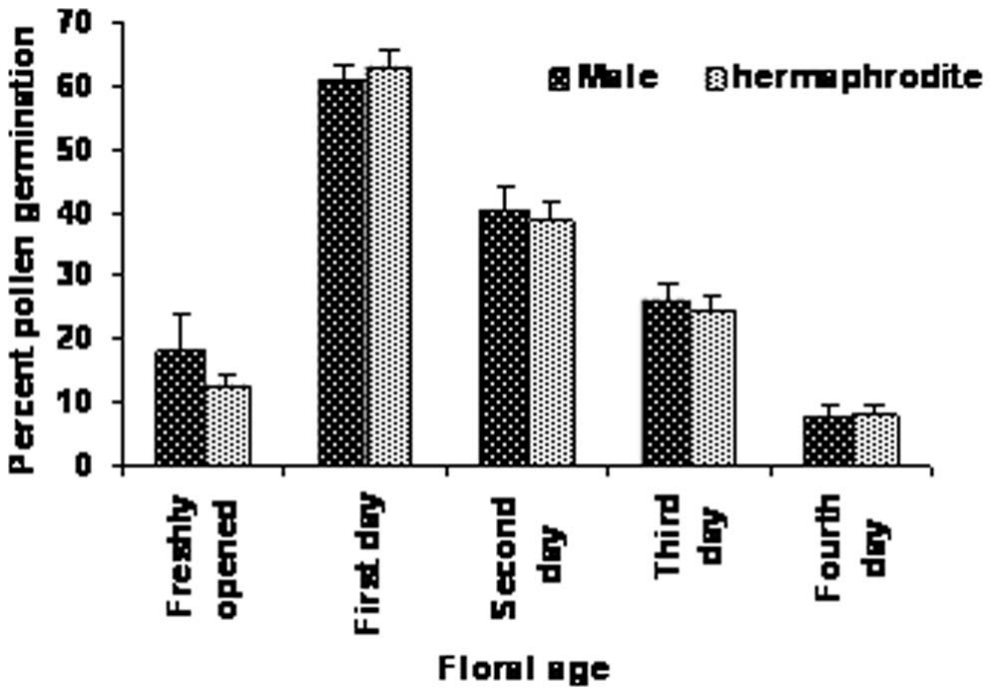
Percentage of pollen germination (male and hermaphrodite) in BK medium throughout the floral life.

**Figure 6:**
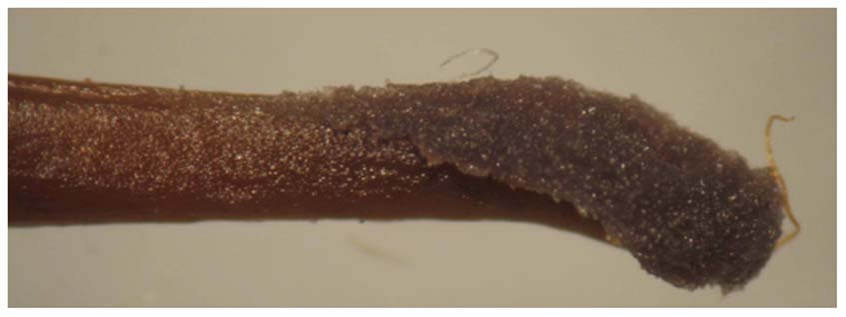
A wet stigma at fully receptive stage.

The hermaphrodite flowers were long-pedicellated and larger than male flowers at anthesis (Figure 3D; Table 3). The anthers were didynamous at full maturity with the longer anther lobes reaching above the receptive surface of the stigma, while the shorter ones remained at the level of the stigma (Figure 4, A–F). The pollen viability percentages were similar to those of male flowers at corresponding developmental stages (Table 2). Flowers opened between 1400–1600 hrs and maximum pollen germination (58–62 percent) was observed in one-day old flowers, and then gradually decreased over time (Figure 5). The pistil in the hermaphrodite flowers was well demarcated into stigma, style and ovary. The flower length at the anthesis was about 10 mm, and the style was straight, green in color, moderately thick and compressed with an oblique stigma. The stigma surface was slightly slanted, papillate with wet sticky exudates (Figure 6). The unilocular superior ovary contained 7.7±0.23 (n = 20) ovules. Detailed floral morphometric data are given in Table 3.

### Nectar production

Flowers started production of nectar when they attained the stage 2 (Table 2). The maximum nectar production was at stage 4 (Table 2) and then gradually decreased and dried up at stage 6. Although the nectar secretion pattern was uniform among different flowers, nectar volume varied significantly (p<0.05) between the two flower morphs (Table 2).

### Pollen morphology

**Figure 7:**
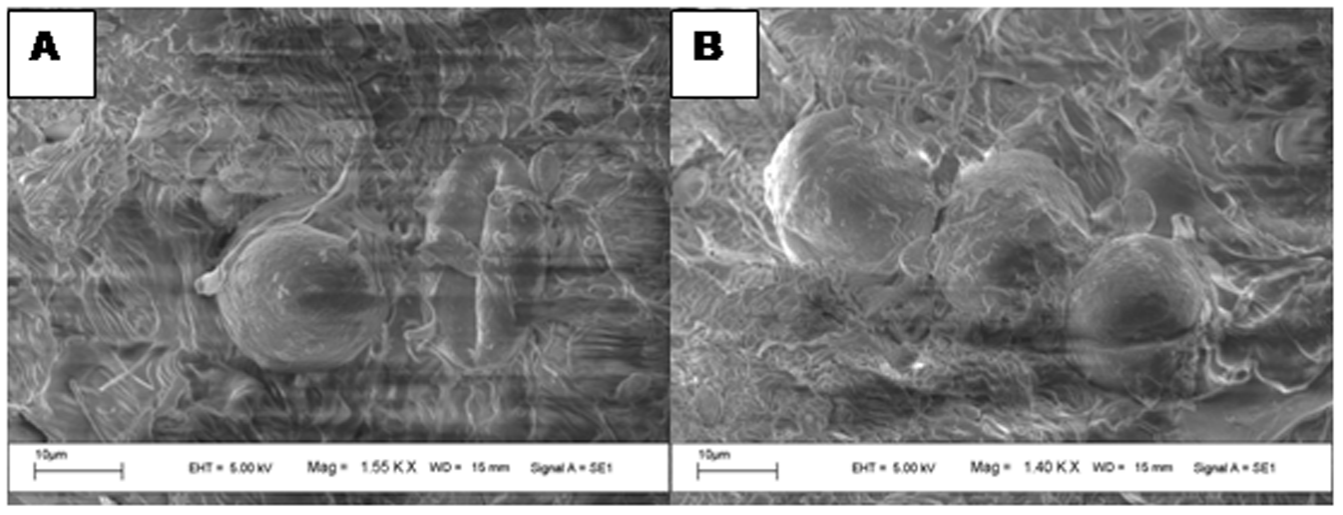
Scanning Electron Micrograph of pollen (A) polar view of male pollen showing polycolpate structure and (B) distal view of hermaphrodite pollen.

Scanning electron microscopic (SEM) observation of the pollen grains of both morphs revealed that pollen grains were of polycolpate (multiple colpi with elongated apertures) and psilate (lacking ornamentation). SEM also revealed that pollen grains of male and hermaphrodite flowers were morphologically similar in size and shape (Figure 7: A, B).

### Mating system

The manual self pollination of hermaphrodites within flowers (autogamous) or between flowers of the same tree (geitonogamous) increased the fruit set considerably as compared to open pollination fruit set (Table 4). The xenogamous pollination of hermaphrodite flowers with pollen from males yielded slightly lower fruit set (not statistically significant) than pollinated with pollen from hermaphrodite flowers. Four out of the nine study sites had only hermaphrodite trees, which produced fruits and seeds consistently under natural conditions suggesting that hermaphrodite flowers set fruits in the absence of male trees in the neighborhood.

**Table 4:** Results of controlled pollination experiments.

Treatment	Number of flowers pollinated	Number of flowers produced fruit	Average Fruit set percentage (± SE)	*Probability level of fruit set
Open pollination	302	146	48.31 (±2.75)	F = 10.325, P<0.001
Spontaneous self pollination	396	157	36.64 (±2.48)	
Hand pollination	143	107	74.91 (±2.92)	
Geitonogamy	160	105	65.59 (±2.82)	
Xenogamy with male	134	78	57.50 (±3.85)	
Xenogamy with hermaphrodite	147	99	68.40 (±3.53)	

*Differences in percent fruit set among treatments were highly significant.

### Pollinators

Altogether nine species of anthophilous insects, which included social bees, beetles and butterflies were collected from flowering *G. assamicus* trees. The social bees were composed of two species namely *Apis cerana* and *Apis dorsata*. *Apis dorsata* was more common than *Apis cerana.* Three species of leaf cutter bees (*Megachile* spp.), one species of beetle and two species of butterflies were also recorded. Among vertebrates, only one nectarivorous bird, the ‘Green-tailed Sunbird or Nepal Yellow-backed Sunbird’ (*Aethopyga nipalensis*) frequently visited flowers during the peak flowering period (Table 5).

**Table 5:** Flower visitors of *G. assamicus.*

Sl. No.	Common name	Order	Family	Scientific name
1	Honey bee	Hymenoptera	Apidae	*Apis cerana* Fabr.
2	Giant honey bee	Hymenoptera	Apidae	*Apis dorsata* Fabr.
3	Bumblebee	Hymenoptera	Apidae	*Bombus* sp.
4	Leaf cutter bees	Hymenoptera	Megachilidae	*Megachile* sp.
5	Leaf cutter bees	Hymenoptera	Megachilidae	*Megachile* sp.
6	Leaf cutter bees	Hymenoptera	Megachilidae	*Megachile* sp.
7	Flower chaffer	Coleoptera	Scarabaeidae	Unidentified
8	Moth/Butter fly	Lepidoptera	Unidentified	Unidentified
9	Moth/Butter fly	Lepidoptera	Unidentified	Unidentified
10	Green-tailed Sunbird	Falconiformes	Accipitridae	*Aethopyga nipalensis*

### Equilibrium male frequency in the population

**Figure 8:**
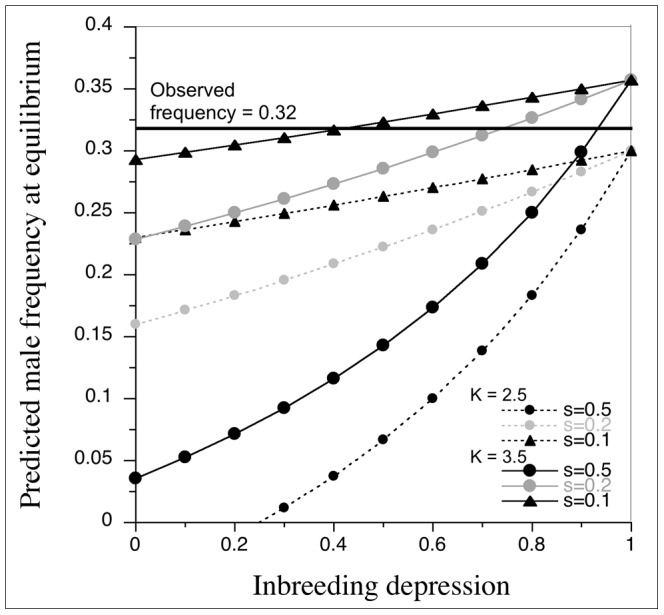
Predicted male frequency versus inbreeding depression. K values slightly below (K = 2.5) or above (K = 3.5) the estimated K value (K = 2.9) based on the observed number of flowers in male and female trees were used. Three selfing rates (s = 0.1, 0.2, and 0.5) were chosen. The observed frequency of male trees in the population was 32.14%.

The observed male frequency was slightly higher than the expected equilibrium frequency of males under theoretical expectations with high level of inbreeding depression (Figure 8).

## Discussion

Although vegetative phenology of male and hermaphrodite trees of *G. assamicus* was similar, male trees start flowering earlier than the hermaphrodites, which provides more pollination opportunities to males [31] as observed in many dioecious plants [32], [33]. In addition, male trees produce almost three time more flowers than hermaphrodite trees increasing the pollination success of male trees and enhances the male fitness [34] and outcrossing rates [35]. Moreover, the observed nearly two-fold increase (97%) in overall nectar production in male trees may play a significant role in attracting pollinators [36] to secure pollination success [37] and increase male fitness [38]. This increased fitness of male trees is crucial for the evolution and maintenance of androdioecy [12], [39] and dioecy [10], [40]. The predicted equilibrium male frequencies under various levels of inbreeding and selfing rates based on Charlesworth & Charlesworth [30] indicated that high level of inbreeding depression is required to maintain the observed frequency of males in the population (Figure 8). The data on flower production over a long duration, empirical selfing rates and the level of inbreeding depression of *G. assamicus* are needed to assess the congruence of observed data with theoretical prediction to infer equilibrium frequency of males in *G. assamicus* populations. Most likely, the realized K value may be higher than the estimated K value based on the number of flowers produced in male and hermaphrodite trees.

The mating system analyses through controlled pollination experiments revealed that hermaphrodite individuals of *G. assamicus* are self-compatible and autogamous in nature. Moreover, four out of nine sites had only hermaphrodite trees, which produced fruits and seeds under natural conditions. This supports the view that hermaphrodite flowers are complete and can set fruit in the absence of male trees. The xenogamous crosses with pollen from male trees resulted in fruit set confirming the fertility of pollen from male trees. Our results from controlled pollination experiments (Table 4) provided convincing evidence that *G. assamicus* individuals are cosexual with both male and female functions as well as co-occurrence of male and hermaphrodite trees in natural populations. This observation is in agreement with the results of well-studied animal androdioecious taxa where mixed populations of males and self-compatible hermaphrodites co-occur [41]–[44]. Our observation of multiple pollinator species in *G. assamicus* trees also suggests that pollinators are relatively non-specialized as in dioecious plants [45] that are pollinated by unspecialized pollinators such as small bees, flies, and other dipteran species [46], [47].

### Evolution and maintenance of androdioecy in *G. assamicus*

Maintenance of an androdioecious breeding system requires complete outcrossing or low selfing rate, and high inbreeding depression [10], [48]. Theoretical modelling has shown that males can invade a cosexual population only if the fitness of males is greater than twice than the cosexual individuals, and a fertility advantage is necessary for the evolution of androdioecy mating system [19], [24]. Thus, the cosexual individuals in *G. assamicus* may have evolved by breakdown of a dioecious ancestral system where females gained some male function. Significantly lower flower production in hermaphrodites than that of males suggests that male function in hermaphrodites are weak. Similar observations in other functionally androdioecious species such as *Datisca glomerata* [41] and *Morinda umbellata* subsp. *boninensis* [26] support these predictions [42]–[44], [49]. The high numbers of flowers on male than hermaphrodite trees, and early flowering of male trees imply that, when males are present, the selfing rate of hermophrodites is probably low. Carlson [50] demonstrated this phenomenon in a protandrous herb *Chrysothemis friedrichsthaiana*.

### Did *G. assamicus* hermaphrodites evolve from a dioecious ancestor?

Androdioecy is considered as an intermediate step towards the evolution of dioecy [10], [39], [40], [51], [52] or may have evolved from dioecy as result of selection for male function in females for reproductive assurance during colonization [24], [25], [53], [54]. In *G. assamicus*, hand pollination increases the fruit set, suggesting pollination limitation for fruit set. Phylogenetic studies in conjunction with mating system analyses of related species are needed to discern if androdioecy in *G. assamicus* evolved from hermaphrodites or dioecious progenitors.
